# IL-18 and NFE2L2/NRF2 in Adult ADHD: An Inflammation–Redox Framework for Salivary Biomarker Research

**DOI:** 10.3390/ijms27219732

**Published:** 2026-10-30

**Authors:** Ion Andrei Hurjui, Ionela Lacramioara Serban, Loredana Liliana Hurjui, Raluca Jipu, Ion Hurjui, Daniela Maria Tanase, Cristina Claudia Tarniceriu, Carina Balcos, Irina Dobrin, Romeo Petru Dobrin

**Affiliations:** 1 Faculty of Medicine, Grigore T. Popa University of Medicine and Pharmacy, 700115 Iasi, Romania; hurjui_ion-andrei@d.umfiasi.ro (I.A.H.); ionela.serban@umfiasi.ro (I.L.S.); ion.hurjui@umfiasi.ro (I.H.); daniela.tanase@umfiasi.ro (D.M.T.); claudia.tarniceriu@umfiasi.ro (C.C.T.); carina.balcos@umfiasi.ro (C.B.); irina.dobrin@umfiasi.ro (I.D.);; 2 Sf. Spiridon County Clinical Emergency Hospital, 700111 Iasi, Romania; 3 Institute of Psychiatry Socola, 36 Bucium Street, 700282 Iasi, Romania

**Keywords:** adult ADHD, IL-18, NFE2L2, NRF2, saliva, inflammation, oxidative stress, inflammasome, salivary biomarkers

## Abstract

Attention-deficit/hyperactivity disorder (ADHD) is a heterogeneous neurodevelopmental disorder, and increasing evidence suggests that inflammatory and oxidative processes may contribute to its biological complexity. Despite these observations, no molecular biomarker has yet been established for clinical diagnosis. In this narrative review, we examine interleukin-18 (IL-18) and the NFE2L2/NRF2 antioxidant pathway as two potentially complementary components of the inflammation–redox landscape in adult ADHD, with particular attention to their possible assessment in saliva. For this purpose, we conducted a focused search of PubMed/MEDLINE, Scopus, and Web of Science, bringing together evidence from human ADHD studies, mechanistic and experimental research, and studies investigating the feasibility of salivary biomarkers. IL-18 is closely associated with inflammasome-mediated inflammatory signaling, whereas NFE2L2 encodes NRF2, a major regulator of antioxidant and cytoprotective responses. The evidence for these two targets in adult ADHD is not equally developed. Direct human data on IL-18 remain limited, while more recent studies have identified alterations in NRF2-related antioxidant defenses in adults with ADHD. Both IL-18 and NRF2 can be measured in human saliva, although the available evidence comes from clinical populations other than ADHD. Their detection in saliva therefore demonstrates analytical feasibility but does not establish their validity as ADHD biomarkers. At present, neither marker has been validated in the saliva of adults with ADHD. Studying them together may nevertheless provide a useful way to explore the relationship between inflammatory activity and antioxidant regulation. Future studies will require standardized saliva collection, careful assessment of oral health, and characterization of systemic inflammatory and metabolic conditions, medication use, and smoking. Initial studies should also include paired saliva and blood samples to determine how salivary measurements relate to circulating levels and whether they capture biologically meaningful variation in adult ADHD.

## 1. Introduction

Attention-deficit/hyperactivity disorder (ADHD) is a neurodevelopmental disorder characterized by persistent symptoms of inattention and/or hyperactivity–impulsivity that interfere with daily functioning. Although ADHD is usually associated with childhood, for many individuals the symptoms continue into adult life, affecting work, relationships, emotional regulation, and quality of life [1]. Diagnosis is still based on clinical assessment. Biological markers, if identified, would therefore be expected to add to this assessment rather than replace it.

Much of what is known about ADHD neurobiology has come from research on dopaminergic and noradrenergic neurotransmission. These pathways remain central to the disorder, but they are unlikely to account for its biological heterogeneity on their own. In recent years, attention has also turned to inflammatory and oxidative processes [2,3,4,5]. Changes in circulating inflammatory markers have been reported, including higher IL-6 levels, although findings vary considerably between studies [2]. This variability is important. It suggests that inflammation may not be a common feature of all individuals with ADHD, but could be more relevant in particular patients or under certain biological conditions.

Oxidative stress has followed a similar pattern. Alterations in systemic oxidative status have been observed in adults with ADHD [3], and meta-analytic findings point toward increased oxidative stress [4]. The literature, however, remains relatively limited and methodologically diverse. More recently, changes in the SIRT-1/NRF2/HO-1 antioxidant defense axis have been reported in adults with ADHD [5]. This raises a somewhat different question from simply asking whether oxidative damage is increased: how effectively can cells respond when they are exposed to redox stress?

This question also brings inflammation into the picture. The two processes are closely linked. Reactive oxygen species (ROS) can promote inflammatory signaling and inflammasome activation, while inflammation itself can increase ROS production and place additional pressure on antioxidant defenses. Looking at these processes together may therefore be more informative than considering each one separately, particularly in a disorder as heterogeneous as ADHD.

IL-18 is one possible target within the inflammatory side of this relationship. A member of the IL-1 cytokine family, IL-18 is produced through proteolytic processing of pro-IL-18 and is closely associated with inflammasome activity [6]. It also has a role in neuroimmune signaling [7]. What is known specifically about IL-18 in adult ADHD, however, is still limited. Inflammatory changes have been described in ADHD more broadly [2,8], but direct human studies of IL-18 in adults with the disorder are scarce. At present, this makes IL-18 an interesting candidate for investigation rather than an established ADHD biomarker.

For the redox component, we focus on nuclear factor erythroid 2 like 2 (NFE2L2), the gene encoding nuclear factor erythroid 2-related factor 2 (NRF2). NRF2 regulates antioxidant and cytoprotective responses through the KEAP1–NRF2 system [9,10]. In this case, there is some direct evidence from adults with ADHD. Gürbüzer et al. found lower serum NRF2 concentrations in adults with ADHD compared with healthy controls, together with an inverse association between NRF2 levels and symptom severity [5]. One study is not enough to establish a consistent pattern, and these findings need to be replicated. Nevertheless, they provide a clinical rationale for examining NRF2-related antioxidant regulation more closely in adult ADHD.

Saliva is particularly attractive for this type of research because it can be collected easily and repeatedly without an invasive procedure. It also contains a wide range of potentially informative molecules, including proteins, peptides, hormones, cytokines, metabolites, and nucleic acids derived from salivary glands, oral tissues, gingival crevicular fluid, and systemic circulation [11,12,13,14,15]. These advantages come with an important limitation: saliva is influenced by local events in the oral cavity. Oral health, salivary flow, collection conditions, time of day, medication, smoking, and other pre-analytical factors can all affect measured concentrations [16,17,18,19,20,21]. Detecting a molecule in saliva is therefore only the first step; it does not show that the molecule is specific to a disease.

These factors are particularly important when studying IL-18 and NRF2, as changes in their salivary levels may reflect influences unrelated to ADHD. Careful consideration of these variables is therefore essential for interpreting future findings in adults with ADHD.

There is already some experience with saliva in ADHD research. Pediatric studies have examined biochemical changes as well as oral and salivary characteristics [22,23]. In adults, salivary cortisol has been used to study stress responses and hypothalamic–pituitary–adrenal-axis activity [24,25]. These studies show that saliva can be used successfully in ADHD research, but they do not tell us whether inflammatory or redox-related salivary markers have diagnostic value.

Why, then, consider IL-18 and NRF2 together? The rationale is not that an IL-18–NRF2 axis has already been demonstrated in adults with ADHD. It has not. Rather, the two targets represent different parts of a connected biological picture. IL-18 is related to inflammasome-associated inflammatory signaling, whereas NRF2 is involved in the adaptive response to oxidative stress. Studying both may provide a way to explore these two sides of immune–redox biology in the same individuals, using the same minimally invasive matrix.

With this distinction in mind, the review has three main aims. First, we separate findings obtained directly in adults with ADHD from mechanistic and experimental evidence involving IL-18 and NFE2L2/NRF2. Second, we examine what existing salivary studies can and cannot tell us about measuring these targets. Third, we consider what would be required methodologically to study IL-18 and NRF2 together in the saliva of adults with ADHD. At present, the evidence is not sufficient to assess their diagnostic accuracy, and we do not attempt to do so here.

While previous reviews have discussed inflammation and oxidative stress in ADHD, these processes have generally been considered as broader biological mechanisms rather than through the specific relationship between IL-18 and NRF2. The rationale for examining these two pathways together lies in their complementary roles: IL-18 reflects inflammasome-related inflammatory signaling, whereas NRF2 regulates cellular antioxidant and cytoprotective responses. Considering them within a shared framework may help explore how inflammatory activation and antioxidant regulation interact in ADHD. This approach is intended to provide a more focused perspective for future research rather than to suggest an established mechanistic relationship or validated biomarker combination in adults with ADHD.

### Literature Search Strategy and Scope of the Review

We searched PubMed/MEDLINE, Scopus, and Web of Science using combinations of the terms “ADHD”, “adult ADHD”, “attention-deficit/hyperactivity disorder”, “IL-18”, “interleukin-18”, “NFE2L2”, “NRF2”, “oxidative stress”, “antioxidant response”, “inflammation”, “neuroinflammation”, “inflammasome”, “saliva”, and “salivary biomarkers”. The search terms were combined using Boolean operators (AND/OR) to explore the relationships between ADHD, inflammatory and redox pathways, and salivary biomarkers. Reference lists of relevant original studies and reviews were also examined to identify additional publications.

Our search focused primarily on human studies involving adults with ADHD. However, we did not identify studies directly investigating salivary IL-18 or NRF2 in this population. We therefore considered additional evidence from mechanistic research, experimental ADHD models, and studies examining these markers in human saliva in other clinical settings. Pediatric studies and research involving individuals without ADHD were included only to provide biological or methodological context, rather than evidence of biomarker validation in adult ADHD.

Studies were selected based on their relevance to adult ADHD, IL-18 or NRF2-related biological pathways, and the feasibility of salivary biomarker assessment. Priority was given to original human studies, while experimental and mechanistic investigations were considered when they provided relevant biological context. Publications without sufficient methodological information or a clear connection to the review objectives were not considered. Study selection was guided by thematic relevance rather than by a formal systematic screening procedure.

Given the differences in study populations, biological matrices, experimental approaches, and reported outcomes, a quantitative synthesis was not appropriate. We therefore adopted a narrative approach, focusing on peer-reviewed publications that provide sufficient methodological detail to support meaningful interpretation of the findings. Grey literature and conference abstracts were excluded because they often lack the methodological detail needed for a reliable assessment.

A formal risk-of-bias assessment was not undertaken because the purpose of this narrative review was to integrate biological and methodological evidence rather than to systematically evaluate individual studies.

Throughout the review, we distinguish between evidence obtained directly from adults with ADHD, findings from mechanistic and experimental studies, and research supporting the feasibility of salivary measurements. This distinction is important because biological plausibility and the detection of a molecule in saliva do not necessarily establish its value as a biomarker of adult ADHD. The review does not aim to systematically compare candidate biomarkers for adult ADHD, but rather to explore the biological rationale and research potential of IL-18 and NRF2, while clearly distinguishing salivary detectability from clinical validation.

## 2. Salivary Biomarkers in Adult ADHD

Saliva is increasingly used in biomedical research because it permits non-invasive, inexpensive, and repeatable sampling. Whole saliva contains proteins, peptides, enzymes, hormones, metabolites, nucleic acids, and immune-related molecules originating from salivary glands, oral tissues, gingival crevicular fluid, and systemic circulation [15,16,17,18]. These properties are advantageous for longitudinal or repeated-measures designs, but the mixed biological origin of salivary constituents also complicates interpretation.

In ADHD, salivary research remains less developed than blood-based biomarker research and is heterogeneous with respect to age, medication exposure, diagnostic characterization, collection procedures, and analytical methods. Much of the available evidence concerns children or adolescents. Consequently, pediatric findings can inform feasibility and study design but should not be treated as direct evidence for adult ADHD.

Neuroendocrine markers illustrate both the potential and the limitations of saliva. In adults with ADHD, Ramos-Quiroga et al. evaluated the cortisol awakening response and reported substantial interindividual heterogeneity and associations with selected clinical dimensions [19]. In children, Wang et al. reported differences in salivary DHEA and the DHEA/cortisol ratio, whereas basal cortisol alone was not significantly associated with ADHD [20]. These findings illustrate the potential value of examining biologically related measures alongside individual analytes, while also underscoring age- and context-dependent variability.

Saliva has also been evaluated for therapeutic drug monitoring. Preiskorn et al. measured methylphenidate and ritalinic acid in paired serum and saliva samples from patients with ADHD, including adults [21]. Salivary methylphenidate correlated with serum concentrations, but interindividual variability and salivary pH affected interpretation [21]. This study is important methodologically because it demonstrates that a measurable salivary analyte may still require matrix-specific validation before it can be interpreted as a surrogate for circulating exposure.

Pre-analytical standardization is therefore essential. Salivary concentrations can vary with stimulated versus unstimulated collection, flow rate, time of day, food and beverage intake, oral hygiene, smoking, medication, hydration, processing, and storage [15,16,22]. Ngamchuea et al. detailed the influence of collection and analytical procedures [15], while Mortazavi et al. documented substantial methodological variation across saliva studies and emphasized the need for standardized collection, transportation, preparation, and storage [22].

Inflammatory and redox biomarkers require additional caution because whole saliva can reflect both systemic biology and local oral processes. Periodontal inflammation, gingival bleeding, oral lesions, and other oral conditions can materially alter measurements of cytokines and oxidative stress [17,23]. Oral examination and periodontal characterization should therefore be incorporated into biomarker protocols rather than treated as optional covariates.

Current evidence supports saliva as a feasible research matrix in ADHD, but it does not establish a clinically validated salivary diagnostic biomarker. Proteomic studies have identified molecular pathways related to inflammation, mitochondrial metabolism, neurotransmission, myelination, and intracellular signaling, yet no salivary molecular marker has achieved established diagnostic utility [24]. This distinction between discovery and validation is particularly important in adult ADHD, where dedicated salivary studies remain limited.

For the present review, saliva is therefore considered a candidate matrix for testing a biological hypothesis, not evidence of biomarker validity in itself. IL-18 and NRF2 are of interest because they represent complementary inflammatory and antioxidant processes and because both have been quantified in human saliva in non-ADHD clinical settings. Whether their salivary concentrations provide reproducible and biologically meaningful information in adults with ADHD remains an empirical question that requires direct investigation.

## 3. IL-18: Inflammation and Neuroinflammation in ADHD

Interleukin-18 (IL-18) is a pleiotropic member of the IL-1 cytokine family involved in both innate and adaptive immune responses. It is initially produced as the inactive precursor, pro-IL-18, and becomes biologically active after proteolytic cleavage, primarily by caspase-1 [25]. This places IL-18 downstream of inflammasome activation, at a point where cellular stress and innate immune signaling can converge.

NLRP3 is one of the most widely studied inflammasomes in inflammatory and neurobiological research. Its activation promotes caspase-1-dependent processing of IL-18, although the biological activity of IL-18 cannot be inferred from its concentration alone. IL-18-binding protein, receptor availability, the cells producing the cytokine, and the surrounding cytokine environment all influence its effects [25]. This becomes particularly relevant when IL-18 is considered as a biomarker. Measuring its concentration in a peripheral sample capture only part of a more complex signaling process.

There is also a biological basis for considering IL-18 in the nervous system. IL-18 and components of its receptor are expressed in neuronal and glial cells, and experimental studies have linked IL-18 signaling to stress responses, neuronal survival, behavior, and inflammatory regulation [26]. This makes a neuroimmune role plausible. It does not, however, mean that IL-18 measured in blood or saliva can be taken as a direct readout of what is happening in the brain.

When ADHD is considered specifically, the evidence becomes much thinner. Reviews of inflammatory biomarkers have reported immune-related changes in ADHD, but the findings are heterogeneous, and no inflammatory marker has achieved established diagnostic use [27]. IL-18 has also received far less direct attention than cytokines such as IL-6. Human data specifically examining IL-18 in adults with ADHD remain scarce. At this point, it is therefore reasonable to investigate IL-18 as part of the broader inflammatory biology of ADHD, but not to describe IL-18 dysregulation as an established feature of the disorder.

Some additional support comes from experimental work on inflammasome signaling. Shan et al. reported that suppressing astrocytic NLRP3 inflammasome activation in an ADHD-like mouse model was accompanied by improvements in behavioral and cognitive abnormalities [28]. Since IL-18 is one of the cytokines associated with downstream inflammasome signaling, this finding gives a mechanistic reason to investigate the pathway further. It does not show, however, that IL-18 itself has a causal role in human ADHD.

The connection with oxidative stress provides another reason to consider IL-18 in this context. ROS and mitochondrial dysfunction can promote inflammasome activation, while inflammatory signaling can itself add to the oxidative burden. IL-18 therefore sits within a broader network in which inflammatory and redox processes can influence one another. This is also why it may be useful to study IL-18 alongside NRF2-regulated antioxidant responses. What the current evidence does not support is a specific IL-18–NRF2 molecular axis in adult ADHD [29,30,31,32].

IL-18 can be measured in human saliva, but the studies available so far come from clinical settings other than ADHD. Techatanawat et al., for example, measured IL-18 in paired saliva and serum samples from individuals with type 2 diabetes, with and without periodontitis [29]. Salivary concentrations differed according to metabolic and periodontal inflammatory status. The study is useful for two reasons: it shows that salivary measurement is feasible, but it also makes clear that both local oral inflammation and systemic disease can affect the result.

A similar picture emerges from other inflammatory conditions. Zhang et al. reported higher IL-18 concentrations in saliva and serum of patients with oral lichen planus, with salivary levels associated with disease severity [30]. Arunachalam et al. examined salivary IL-18 alongside AIM2 and IFI16 in participants with periodontitis, diabetes, or both and found differences among the clinical groups [31]. These studies confirm that IL-18 is detectable in saliva, but they also underline a practical problem for ADHD research: oral health can substantially influence the same measurement we would be trying to interpret as part of a broader inflammatory profile.

For this reason, salivary IL-18 in adults with ADHD could not simply be interpreted as a marker of neuroinflammation. Periodontal disease, gingival inflammation or bleeding, oral lesions, smoking, metabolic disease, medication exposure, and other inflammatory conditions may all contribute to the measured concentration. Any study comparing adults with and without ADHD would need to characterize these factors carefully and use a standardized sampling protocol before differences between groups could reasonably be linked to ADHD-related biology.

At present, the argument for studying salivary IL-18 in adult ADHD comes from several pieces of evidence that answer different questions. We know a good deal about IL-18 biology and its neuroimmune relevance [25,26]. There is broader evidence of inflammatory changes and inflammasome involvement relevant to ADHD [27,28]. We also know that IL-18 can be measured in saliva in other inflammatory conditions [29,30,31]. What is still missing is the step that connects these observations directly: studies measuring salivary IL-18 in well-characterized adults with ADHD, Table 1. Until such case–control and longitudinal data are available, salivary IL-18 should remain a candidate for investigation rather than an ADHD-associated biomarker.

## 4. NFE2L2: Oxidative Stress and Antioxidant Response in ADHD

Oxidative stress develops when the production of reactive oxygen and nitrogen species exceeds the capacity of the systems that normally control or repair oxidative damage. Interest in this process has grown in ADHD research. A recent meta-analysis of studies published between 2010 and 2025 found evidence of increased oxidative damage and reduced antioxidant capacity across age groups and biological matrices [32]. The results were not uniform across studies; however, this suggests that oxidative stress is unlikely to represent a single biological signature of ADHD. It may be more useful to view it as one dimension of the disorder’s biological heterogeneity.

One of the main regulators of the cellular response to oxidative stress is nuclear factor erythroid 2-related factor 2 (NRF2), encoded by the NFE2L2 gene [33,34]. Under normal conditions, NRF2 is tightly controlled by Kelch-like ECH-associated protein 1 (KEAP1), which promotes its degradation. When cells are exposed to oxidative or electrophilic stress, redox-sensitive residues in KEAP1 are modified. NRF2 can then stabilize, accumulate, and move into the nucleus [33].

Once in the nucleus, NRF2 binds to antioxidant response elements (AREs) and regulates genes involved in glutathione metabolism, detoxification, redox balance, and cellular protection. These include heme oxygenase-1 (HO-1), NAD(P)H quinone oxidoreductase 1 (NQO1), and the glutamate–cysteine ligase subunits [33,34]. This distinction matters when NRF2 is discussed as a potential biomarker. NRF2 is part of the cellular response to oxidative stress; it is not simply another measure of oxidative damage.

This regulatory role has also made NRF2 relevant to neuropsychiatric research. NRF2 signaling interacts with mitochondrial function, inflammatory pathways, and mechanisms involved in cellular resilience [34]. In ADHD, an impaired antioxidant response could, in principle, leave cells more vulnerable to oxidative stress. Whether this actually occurs in the disorder and how important it may be clinically requires ADHD-specific evidence.

Some clues come from experimental models. Abu-Elfotuh et al. examined an ADHD-like rat model and reported changes in the NRF2/HO-1 pathway alongside TLR4/NF-κB/NLRP3/caspase-1 inflammatory signaling [35]. The study is relevant because antioxidant and inflammasome-related pathways changed within the same experimental setting. It cannot tell us, however, whether the same molecular pattern is present in adults with ADHD.

Another experimental study brings dopamine into this picture. In the spontaneously hypertensive rat model of ADHD, Ding et al. linked impaired NRF2/KEAP1/HO-1 signaling with changes in antioxidant capacity, glutathione status, and dopamine homeostasis [36]. Activation of the pathway was associated with an improvement in DAT–VMAT2 balance [36]. This is particularly interesting in the context of ADHD because it connects redox regulation with dopaminergic biology rather than treating oxidative stress as an isolated process. As with other animal findings, its relevance to human ADHD still needs to be demonstrated.

There is a biological basis for such an interaction. Dopamine metabolism and intracellular dopamine handling are sensitive to the redox environment. Mitochondrial dysfunction, altered vesicular storage, transporter activity, and dopamine metabolism can affect ROS generation; excessive oxidative stress can, in turn, influence membrane proteins and neuronal energy metabolism. NRF2-dependent antioxidant regulation may therefore be one of the mechanisms linking cellular redox capacity with catecholaminergic homeostasis [34,36].

At this point, NRF2 differs from IL-18 in one important respect: there is already some direct evidence from adults with ADHD. Gürbüzer et al. reported lower serum concentrations of NRF2, SIRT-1, and HO-1 in adults with ADHD compared with healthy controls. They also found an inverse association between NRF2 concentrations and ADHD symptom severity [5]. These findings are clinically relevant, but they come from an emerging evidence base and need independent replication. They provide a reason to investigate NRF2 further, not evidence that NRF2 has diagnostic value in ADHD.

Overall, the literature gives a reasonable basis for studying NRF2-related antioxidant regulation in adult ADHD. The argument does not depend on NRF2 already being an ADHD biomarker. Rather, it comes from several converging observations: the broader evidence of oxidative imbalance in ADHD, the established role of NRF2 in antioxidant regulation, findings from experimental ADHD models, and the first direct observations in adults with the disorder [5,32,33,34,35,36]. A different question is whether any of these can be captured reliably in saliva.

There are some reasons to explore that possibility. Wati et al. showed experimentally that inhibiting KEAP1 and activating the NRF2 pathway reduced oxidative stress and age-related pathological changes in salivary glands [37]. This confirms that the KEAP1–NRF2 system is functionally relevant in salivary tissue. It does not mean, however, that NRF2 measured in saliva necessarily reflects NRF2 activity elsewhere in the body or in the central nervous system.

Human data provide a second piece of the puzzle. Yildiz et al. measured NRF2 protein by ELISA in paired saliva and serum samples from patients with stage III periodontitis and healthy controls [38]. Their study shows that NRF2 protein can be quantified in human saliva. The clinical setting is equally informative, because periodontitis involves local inflammatory and oxidative processes that may themselves affect salivary NRF2 concentrations [38]. Detectability, in other words, does not resolve the question of biological origin.

What is still missing is direct evidence from adults with ADHD. No study included in this review has validated salivary NRF2 in this population. At present, we know that oxidative imbalance has been reported in ADHD [32], that NFE2L2/NRF2 has a well-established role in redox regulation [33,34], that experimental ADHD models provide mechanistic support [35,36], that initial serum findings are available in adults with ADHD [5], and that NRF2 can be measured in human saliva in another clinical setting [38], Table 2. These observations make salivary NRF2 worth investigating, but they do not answer the central question. Whether salivary NRF2 is reproducible, biologically informative, or related to ADHD-specific features will have to be tested directly.

## 5. IL-18 and NFE2L2/NRF2 at the Inflammation–Redox Interface

Inflammation and oxidative stress are closely interconnected. Reactive oxygen species (ROS) can disrupt mitochondrial function and promote inflammatory signaling, including inflammasome activation. Inflammatory responses, in turn, may increase ROS generation and place additional pressure on antioxidant defenses [39,40]. In neuropsychiatric disorders, these processes may contribute to disturbances in cellular homeostasis even in the absence of classical systemic inflammation.

IL-18 and NRF2 occupy different positions within this biological landscape. IL-18 is associated with inflammasome-related inflammatory activation, whereas NRF2 coordinates an adaptive response to oxidative and electrophilic stress. They should not be viewed as components of a demonstrated linear molecular pathway. Their interest here comes from the possibility that they capture different aspects of the same broader biological context: inflammatory activity on one side and antioxidant regulation on the other.

### 5.1. Mechanistic Crosstalk Between Inflammatory and Redox Pathways

Oxidative stress can promote NLRP3 inflammasome activation through mitochondrial dysfunction, cellular damage, and ROS-dependent signaling. Once activated, the inflammasome promotes caspase-1-dependent maturation of pro-IL-18, linking cellular stress with IL-18-mediated inflammatory responses [39,40]. The relationship can also operate in the opposite direction, as inflammatory activation may further increase oxidative burden.

NRF2 is relevant to this interaction because its antioxidant and cytoprotective functions can alter the cellular environment in which inflammasome signaling occurs. Hennig et al. and Jhang and Yen describe extensive crosstalk between NRF2 signaling and inflammasome activation, with predominantly antagonistic effects reported in several experimental systems [39,40]. This provides evidence for interaction between redox regulation and inflammasome signaling in general, but not for a specific IL-18–NRF2 molecular pathway in adult ADHD.

One way of approaching these findings is through the concept of immune–redox balance. If NRF2-mediated adaptation is insufficient, oxidative and mitochondrial stress may persist and create conditions that favor inflammatory signaling. A more effective antioxidant response could have the opposite effect. From this perspective, IL-18 and NRF2 may provide complementary insights into inflammatory activity and antioxidant adaptation. Whether this pattern can actually be demonstrated in adults with ADHD remains an open question. Thus, the proposed framework reflects biological plausibility and potential associations rather than an established causal relationship between IL-18, NRF2, and adult ADHD.

### 5.2. Relevance to Adult ADHD

Neither inflammation nor oxidative stress offers a single biological explanation for ADHD. Both, however, have been implicated in at least some ADHD populations, with considerable variability between studies [2,3,4,5,32,41]. Corona discussed the possibility that oxidative stress and neuroinflammation interact in ADHD, with potential consequences for mitochondrial function and neuronal homeostasis [41]. This provides useful context for the present framework, but does not imply that ADHD itself should be regarded primarily as an inflammatory or oxidative-stress disorder.

The available human evidence concerning peripheral inflammatory and redox alterations in adult ADHD remains heterogeneous and does not support a consistent biomarker profile [2,3,4,5,32]. Although some studies have reported increased inflammatory activity or oxidative imbalance, the magnitude and direction of these changes vary across investigations [2,3,4,32]. Such inconsistencies may be related to differences in sample characteristics, clinical heterogeneity, medication exposure, comorbid conditions, and the biological matrices and analytical methods employed [2,32]. Furthermore, inflammatory and oxidative alterations may reflect broader physiological or metabolic processes rather than mechanisms specific to ADHD. These observations suggest that peripheral inflammatory–redox markers should be interpreted within a multifactorial biological framework, rather than as isolated indicators of the disorder. Importantly, the limited direct evidence concerning IL-18 and the emerging findings related to NRF2 do not yet allow definitive conclusions regarding their clinical relevance in adults with ADHD [5,25,26,27,28].

Clinical heterogeneity should also be considered, as adults with ADHD may differ in symptom presentation, severity, and associated conditions. Whether these differences affect IL-18 or NRF2 levels remains unclear and warrants exploration in future studies before drawing conclusions about their biomarker potential.

Persistent oxidative stress and mitochondrial dysfunction can affect cellular energy availability, membrane integrity, and synaptic processes. Cytokine and glial signaling may also modify the neuronal microenvironment and influence oxidative burden [26,34,41]. These mechanisms make an interaction between inflammatory activity and antioxidant capacity biologically plausible. What remains missing is evidence that peripheral or salivary measurements of these processes correspond to the neuronal mechanisms underlying ADHD.

Catecholaminergic neurotransmission offers another possible point of connection. Dopamine and norepinephrine are central to ADHD neurobiology, and experimental work has linked NRF2-regulated redox signaling with DAT–VMAT2 homeostasis [36]. The finding is relevant because it connects redox regulation with dopamine handling in an ADHD-like experimental model. It remains uncertain whether peripheral IL-18, particularly salivary IL-18, or NRF2 carries information about these neuronal processes in adults with ADHD.

For this reason, the interest in IL-18 and NRF2 is not based on their proposed use as diagnostic markers. A more immediate question is whether studying them together could reveal an inflammation–redox dimension that would be missed when individual analytes are considered separately. This question fits within a much broader ADHD biomarker literature in which numerous biochemical and physiological candidates have been explored, but none has yet provided a sufficiently reliable peripheral diagnostic marker [42].

Adults with ADHD are also an informative population in which to examine this question. Age, cumulative treatment exposure, smoking, metabolic factors, comorbidities, sleep, and lifestyle may contribute to biological variability, while several of these factors can also influence inflammatory and redox measurements. Careful phenotyping and appropriate adjustment for these variables will therefore be important when moving beyond simple case–control comparisons.

### 5.3. Translational Potential of Combined IL-18 and NRF2 Assessment

There is already evidence that inflammatory and redox-related markers can be assessed concurrently in saliva. Nam et al. measured salivary CRP, IL-1β, IL-6, and TNF-α, along with 8-OHdG, malondialdehyde, and total antioxidant capacity, in healthy adults, reporting acceptable reproducibility for many of these measurements [43]. Although this does not address ADHD, it shows that several components of inflammatory and oxidative biology can be examined within the same salivary sample.

The range of inflammatory mediators detectable in saliva is also well documented. Prasad et al. reviewed inflammatory biomarkers measurable in saliva and urine [44], while Dongiovanni et al. discussed salivary cytokines, chemokines, acute-phase proteins, and oxidative mediators in the context of chronic inflammatory processes [45]. The challenge is not simply whether these molecules can be measured, but what their concentrations represent, particularly when local oral inflammation may contribute to the observed signal.

The same concern applies to oxidative-stress measurements. Wang et al. described substantial variation in sampling procedures, analytical methods, study populations, and oral conditions across salivary oxidative-stress studies [46]. This is especially relevant when considering NRF2. As a regulatory protein, NRF2 cannot be automatically interpreted in the same way as an oxidative damage product or a global measure of antioxidant capacity.

Experience from neurological biomarker research adds another layer to this discussion. Farah et al. and Maciejczyk et al. describe the growing use of saliva in neurological and redox-related biomarker studies [47,48]. Saliva is therefore a reasonable matrix in which to explore biological processes relevant to the nervous system. What these studies cannot tell us is whether salivary concentrations mirror those in the central nervous system or whether they capture disease-specific mechanisms.

The combined assessment of IL-18 and NRF2 should therefore begin as an empirical question. Do their salivary concentrations, considered together, relate to ADHD status, symptom dimensions, or other clinically relevant variables once oral, metabolic, inflammatory, behavioral, and treatment-related factors are taken into account? That question can be tested directly. Ratios, composite scores, or putative biological subgroups would need to emerge from the data and undergo internal validation before being tested in independent populations.

### 5.4. Limitations, Knowledge Gaps, and Future Perspectives

**Figure 1:**
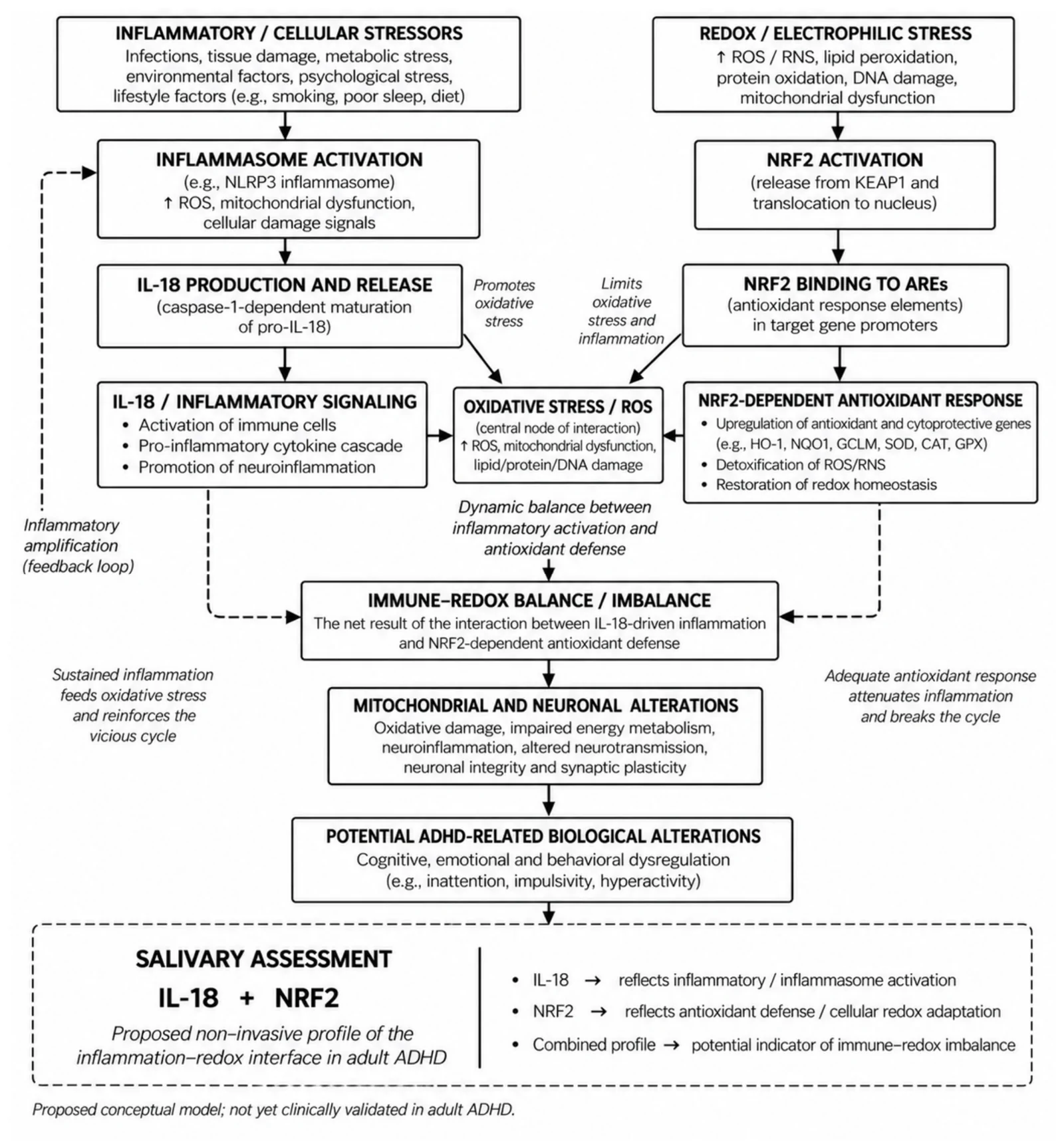
Proposed conceptual model. The suggested links between IL-18, NRF2, and ADHD-related alterations remain hypothetical and have not been validated in adults with ADHD.

The most obvious gap is also the most important one: no direct evidence currently shows that salivary IL-18, salivary NRF2, or their combination distinguishes adults with ADHD from controls, Figure 1. The evidence is uneven across the two markers. For NRF2, there are emerging serum findings directly from adults with ADHD [5]. The case for IL-18 relies more heavily on inflammasome biology and mechanistic evidence [25,26,27,28]. Evidence that either target can be investigated in saliva comes from studies conducted outside ADHD [29,30,31,38]. These different levels of evidence need to remain separate when interpreting the proposed framework.

Saliva introduces another difficulty because it reflects both systemic and local influences. Periodontal inflammation, gingival bleeding, oral lesions, smoking, diet, hydration, salivary flow, circadian timing, medication, and collection procedures can all affect inflammatory or redox measurements [15,16,22,23,43,46]. Blood contamination can further influence selected salivary biomarkers [43]. An oral examination, including periodontal assessment, should consequently be part of future study protocols, rather than addressing oral health only through self-report.

It is equally unclear how closely salivary IL-18 or NRF2 corresponds to blood concentrations. Either marker could reflect circulating concentrations, local glandular or oral processes, or some combination of these sources. This has to be established separately for each analyte. Early studies would benefit from paired saliva and blood collection, together with reporting of assay sensitivity, detection limits, intra- and inter-assay variability, within-person variation, and reproducibility across repeated samples [43,46].

An initial validation study could address several of these uncertainties at once. Adults with well-characterized ADHD and appropriately matched controls should be assessed using clearly documented diagnostic methods and measures of symptom severity. Medication exposure, smoking, body mass index or metabolic status, inflammatory comorbidities, sleep-related factors, and oral and periodontal health should be recorded prospectively. Saliva collection would need a standardized time and a predefined stimulated or unstimulated protocol, with flow rate and pre-analytical handling documented. Paired serum or plasma measurements could clarify cross-matrix relationships, while repeated sampling would provide information on intra-individual stability and possible changes with treatment or symptom variation.

At this stage, establishing whether a reproducible biological signal exists is more important than attempting diagnostic classification. Questions of diagnostic performance, predictive models, or composite inflammation–redox indices become meaningful only after analytical validity, reproducibility, confounding, and clinical associations have been established. The IL-18–NRF2 framework is useful precisely because these propositions can be tested—and, if the data do not support them, rejected.

## 6. Conclusions

IL-18 and NFE2L2/NRF2 offer two different perspectives on the inflammation–redox biology that may be relevant to adult ADHD. The evidence behind them, however, is not equally developed. For IL-18, the rationale comes mainly from its established role in inflammasome and neuroimmune signaling and from broader inflammatory and experimental findings related to ADHD [25,26,27,28]. NRF2 has a somewhat stronger connection with adult ADHD, as mechanistic and experimental findings are now accompanied by initial evidence from serum studies in adults with the disorder [5]. This difference needs to be kept in mind when the two markers are considered together.

Both IL-18 and NRF2 have been measured in human saliva, although not in adults with ADHD [29,30,31,38]. This makes saliva an interesting matrix for further study, particularly because it enables repeated, minimally invasive sampling. At the same time, neither marker has been validated as a salivary biomarker of adult ADHD. Oral inflammation, systemic and metabolic conditions, medication, smoking, and differences in sample collection and handling may all influence the measurements. Being able to detect IL-18 or NRF2 in saliva is therefore an important starting point, but it does not tell us whether either marker is specific or clinically useful in ADHD.

The next step is to study these markers directly in well-characterized adults with ADHD. Such studies will need standardized saliva collection, careful oral and periodontal assessment, appropriate control of relevant confounders, and, at least initially, paired saliva and blood measurements. If consistent associations emerge, studying inflammatory and antioxidant responses together may help describe part of the biological heterogeneity seen in adult ADHD. For now, the value of the IL-18–NRF2 approach lies in providing a biological question that can be tested directly, rather than a diagnostic model that has already been established. Future studies should compare salivary IL-18 and NRF2 levels between adults with ADHD and matched controls, assess their relationship with clinical characteristics, and determine how oral health, medication, and other confounding factors influence these measurements. Such studies would help establish whether these markers have clinical relevance beyond their biological plausibility.

## Figures and Tables

**Table 1:** Evidence supporting the investigation of IL-18 as a salivary biomarker in adult ADHD.

Evidence Domain	Study/Model	Biological Matrix or System	Main Finding	Relevance to Adult ADHD
IL-18 biology	Kaplanski, 2018 [25]	Systemic immune system	IL-18 is an IL-1-family cytokine processed by caspase-1 and involved in innate and adaptive inflammatory responses	Establishes the biological basis for IL-18 as an inflammatory marker
CNS and neuro-inflammation	Alboni et al., 2025 [26]	CNS, neurons and glial cells	IL-18 and its receptors are expressed in the brain and may modulate neuroinflammation, neuronal function, behavior, and stress responses	Supports biological plausibility of IL-18 involvement in neuropsychiatric disorders
ADHD inflammation	Park, 2022 [27]	Human ADHD literature	Inflammatory alterations have been reported in ADHD, although results are heterogeneous and no inflammatory biomarker is diagnostically established	Places IL-18 within the broader inflammatory hypothesis of ADHD
ADHD-related inflammasome signaling	Shan et al., 2026 [28]	Experimental ADHD-like mouse model	Inhibition of astrocytic NLRP3 inflammasome activation improved ADHD-like behavioral and cognitive alterations	Provides mechanistic evidence linking inflammasome activity with ADHD-like phenotypes
Salivary IL-18	Techatanawat et al., 2020 [29]	Saliva and serum	IL-18 was measurable in both matrices and varied according to diabetes and periodontal inflammatory status	Demonstrates feasibility but also highlights oral/systemic inflammatory confounding
Salivary IL-18	Zhang et al., 2012 [30]	Saliva and serum	IL-18 was elevated in saliva and serum of patients with oral lichen planus and associated with disease severity	Demonstrates responsiveness of salivary IL-18 to inflammatory disease
Salivary inflammasome-related biomarkers	Arunachalam et al., 2024 [31]	Saliva	Salivary IL-18, AIM2, and IFI16 differed according to periodontal and metabolic inflammatory status	Supports salivary detection of inflammasome-related pathways while emphasizing oral-health confounding

The evidence presented ranges from mechanistic and experimental findings to human studies of salivary detectability, without establishing clinical validation in adult ADHD.

**Table 2:** Evidence supporting investigation of the NFE2L2/NRF2 pathway and potential salivary NRF2 assessment in adult ADHD.

Evidence Domain	Study	Model/Matrix	Main Finding	Relevance to Adult ADHD
Oxidative imbalance in ADHD	Amiri et al., 2026 [32]	ADHD studies; meta-analysis	Increased oxidative damage markers and reduced antioxidant capacity were reported across ADHD studies.	Establishes the redox background supporting investigation of NFE2L2.
NFE2L2 biology	Yamamoto et al., 2018 [33]	Molecular/cellular evidence	KEAP1 regulates the NFE2L2-encoded antioxidant response through redox-sensitive control and ARE-dependent gene expression.	Establishes NFE2L2 as a central regulator of antioxidant defense.
Neuropsychiatric relevance	Morris et al., 2021 [34]	Neuropsychiatric literature	NFE2L2-related signaling is relevant to oxidative/nitrosative stress, mitochondrial dysfunction and neuroinflammation.	Links the NFE2L2 axis with brain resilience and neuroprotection.
NFE2L2-related signaling in ADHD-like phenotype	Abu-Elfotuh et al., 2022 [35]	Experimental ADHD-like rat model	The NRF2/HO-1 antioxidant axis was modulated together with NLRP3-related inflammatory signaling.	Provides experimental evidence for interaction between NFE2L2-regulated antioxidant and inflammatory pathways.
NFE2L2 and dopamine homeostasis	Ding et al., 2025 [36]	Spontaneously hypertensive rat ADHD model	Activation of the NRF2/Keap-1/HO-1 pathway improved oxidative balance and DAT-VMAT2 homeostasis.	Links the NFE2L2-regulated antioxidant axis with dopaminergic mechanisms relevant to ADHD.
NRF2 signaling in salivary tissue	Wati et al., 2020 [37]	Experimental salivary gland model	KEAP1 inhibition activated the NFE2L2-related pathway and reduced oxidative stress and glandular pathology.	Demonstrates functional relevance of NFE2L2-regulated antioxidant signaling in salivary glands.
Salivary measurement	Yildiz et al., 2026 [38]	Human saliva and serum	The NRF2 protein was quantified in human saliva by ELISA.	Provides analytical evidence supporting salivary assessment while highlighting oral-health confounding.

Note: Direct validation of salivary NRF2 protein in adults with ADHD is currently lacking. The framework integrates mechanistic, experimental, clinical, and salivary evidence without implying that it has established diagnostic validity. The evidence includes experimental findings, preliminary clinical observations, and studies of salivary detectability, which should not be considered equivalent levels of validation.

## Data Availability

No new data were created or analyzed in this study. Data sharing is not applicable to this article.
